# Effect of bone marrow mesenchymal stem cells on experimental pulmonary arterial hypertension

**DOI:** 10.3892/etm.2012.691

**Published:** 2012-08-31

**Authors:** ZHAO-HUA ZHANG, YAN LU, YUN LUAN, JING-JIE ZHAO

**Affiliations:** 1 Department of Pediatrics, The Second Hospital of Shandong University, Jinan;; 2 Department of Pathology, Hospital of Beijing University of Aeronautics and Astronautics, Beijing;; 3 Central Research Laboratory;; 4 Clinical Molecular Biology Laboratory, The Second Hospital of Shandong University, Jinan, P.R. China

**Keywords:** bone marrow mesenchymal stem cells, pulmonary arterial hypertension, pulmonary vascular wall

## Abstract

The aim of the present study was to investigate the effect of bone marrow mesenchymal stem cell (BMSC) transp1antation on lung and heart damage in a rat model of monocrotaline (MCT)-induced pulmonary arterial hypertension (PAH). The animals were randomly divided into 3 groups: control, PAH and BMSC implantation groups. Structural changes in the pulmonary vascular wall, such as the pulmonary artery lumen area (VA) and vascular area (TAA) were measured by hematoxylin and eosin (H&E) staining, and the hemodynamics were detected by echocardiography. Two weeks post-operation, our results demonstrated that sublingual vein injection of BMSCs significantly attenuated the pulmonary vascular structural and hemodynamic changes caused by pulmonary arterial hypertension. The mechanism may be executed via paracrine effects.

## Introduction

Pulmonary arterial hypertension (PAH) is a progressive disorder characterized by abnormally high blood pressure in the pulmonary artery caused by functional and structural alteration to the pulmonary vasculature resulting in an increase in pulmonary vascular resistance (1). Numerous therapies have been proven useful in decreasing pulmonary arterial pressure, but an effective therapy for the long-term outcome in this disorder is lacking (2–4). Monocrotaline (MCT), a pyrrolizidine alkaloid extracted from the seeds of *Crotalaria spectabilis*, used in the model of MCT-induced pulmonary hypertension is known to represent similar pathology to that of primary PAH (5).

Bone marrow mesenchymal cells (BMSCs) are multipotent progenitor cells derived from fetal bone marrow, which have the ability to differentiate into bone, cartilage, muscle, bone marrow stroma, endothelial cells, vascular smooth muscle cells (6,7) and other connective tissues. Studies also suggest that BMSCs secrete a variety of growth factors, such as vascular endothelial growth factor (VEGF) (8,9). Recently, BMSC transplantation has become a potential therapy for PAH.

## Materials and methods

### Animals

A total of 30 healthy adult Sprague-Dawley (SD) rats weighing 200–250 g were purchased from The Animal Experimental Center of Shandong University, China. The study protocol was reviewed and approved by the Institutional Animal Care and Use Committee, The Second Hospital of Shandong University, and the experiments were conducted according to the Guidelines of the American Physiological Society.

### Isolation, culture, immunophenotype analysis and labeling of BMSCs

Bone marrow cells were isolated by flushing the cavity of femurs and tibias, and transferred to a tissue culture dish 90 mm in diameter (10,11). To separate BMSCs and other cells, the Ficoll (1.077) density gradient centrifugation method was used. The white layer composed of mononuclear cells from the upper layer and interface was carefully collected and washed three times. Flow cytometric immunophenotyping was performed using methods described previously (12,13). Briefly, 5×10^5^ cells were suspended with trypsin and were washed twice in phosphate-buffered saline (PBS). Cells were stained with antibodies against CD44, CD29, CD34 and CD90 (BD Biosciences, Franklin Lakes, NJ, USA) for 30 min at 4°C. The percentage of positive cells was determined by fluorescent activated cell sorting (FACS) analysis. Prior to implantation, the cells were labeled with the cross-linkable membrane dye CM-DiI (2 μg/ml, Invitrogen Corp., Carlsbad, CA, USA) (14). The male SD rats were randomly assigned to 3 groups (n=10 in each group) as follows: control group, animals that received a sublingual vein injection of 0.9% saline instead of BMSCs; PAH group, animals that received a subcutaneous injection of 50 mg/kg MCT; BMSC group, animals that received 5×10^5^ labeled BMSC implantation one week after MCT injection.

### BMSC transplantation

One week after MCT injection, the rats were anesthetized by subcutaneous injection of pentobarbital, 5×10^5^ CM-DiI labeled BMSCs were resuspended in 100 μl saline and administered via the sublingual vein.

### Establishment and evaluation of the pulmonary arterial hypertension model

MCT was prepared as described (2) and was dissolved in 1 N HCl, neutralized to pH 7.4 with 0.5 N NaOH, and diluted with saline before injection. Male SD rats (n=10) were randomly assigned to 2 groups as follows: PH group, animals that received a subcutaneous injection of 50 mg/kg MCT; control group, animals that received a subcutaneous injection of 0.9% saline instead of MCT. One week after MCT injection, the rats were anesthetized and fitted with a 3F-Millar Mikro-Tip catheter via the right jugular vein into the right ventricle to obtain base line measurements of hemodynamics, including right ventricular systolic pressure (RVSP), mean right ventricular pressure (MRVP) and mean pulmonary arterial pressure (MPAP). The rats were sacrificed after hemodynamic measurements, and the lung and heart were quickly harvested and fixed *in situ* via the trachea cannula with buffered 4% formaldehyde, and embedded in paraffin. The sections were cut into 4–5 μm slices and were stained with streptavidin peroxidase and hematoxylin and eosin (H&E). The structural changes in the pulmonary vascular wall were observed by immunofluorescence microscopy and wall thickness (MT), blood vessel diameter (ED), pulmonary artery lumen area (VA) and vascular area (TAA) were measured to calculate MT% (=MT/ED) and VA% (=VA/TAA). The ratio of right ventricular weight to body weight (RV/BW) was calculated to measure right ventricular hypertrophy. Results of the hemodynamic parameters, right ventricular hypertrophy, and pulmonary arterial pathological changes were used to evaluate whether the model of pulmonary arterial hypertension was successfully established.

### Immunological and immunohistochemical analysis

Two weeks after BMSC transplantation the rats were anesthetized and the lungs were inflated with OCT compound and quickly frozen in liquid nitrogen and stored at −80°C. Sections were cut into 4 μm slices and fixed in acetone for 10 min at −20°C. The survival of BMSCs was demonstrated by observing the presence of DiI-labeled cells. Immunofluorescence was then performed with goat anti-mouse monoclonal surfactant associated protein C (SP-C, 1:100) IgG antibody, rabbit anti-human von Willebrand Factor (vWF, 1:100) antibody and VEGF (1:100) antibody. FITC-conjugated antiserum was used as a secondary antibody.

## Results

### The analysis of MCT-induced hemodynamics and RV weight

One week after MCT injection, RVSP, MRVP and MPAP were significantly elevated in the PAH group compared with the control (P<0.05), and the ratio of RV/BW was significantly higher in the PAH group compared with the control group (0.49±0.03 vs. 0.57±0.06, P<0.05). These results indicated that MCT led to severe right ventricular hypertrophy and the PAH model was successfully established (Table I).

### Analysis of MCT-induced structural changes in the pulmonary artery wall

**Figure 1:**
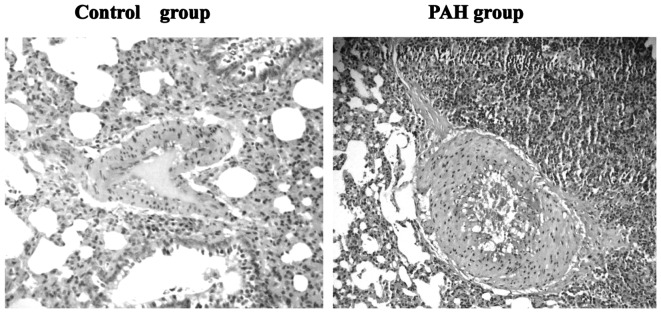
Change in the pulmonary artery wall one week after injection of MCT as determined by H&E staining (x100 magnification). MCT, monocrotaline; H&E, hematoxylin and eosin.

H&E staining demonstrated significant intima thickening and luminal stenosis in the PAH group compared with the control group. The MT% of muscular arteries was significantly increased and the VA% was significantly decreased in the PAH group compared with control group (P<0.01, Table II, Fig. 1).

### MCT-induced lung morphological observation

**Figure 2:**
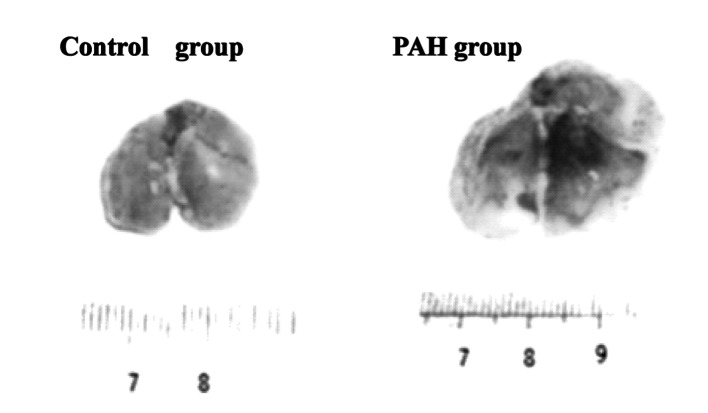
Morphological observation of the lung one week after injection of MCT. MCT, monocrotaline; PAH, pulmonary arterial hypertension.

Two weeks after operation, gross observation showed that enlarged volume, purple color, uneven surface, poor elasticity and partial visible blood stasis were present in the PAH group compared with the normal control group (Fig. 2).

### Effect of BMSCs on hemodynamics and RV impairment

Two weeks after BMSC administration, RVSP, MRVP and MPAP were significantly lower in the BMSC group compared with the PAH group, the ratio of RV/BW was significantly lower in the BMSC group compared with the PAH group (P<0.05, Table III).

### Effect of BMSCs on pulmonary artery wall

**Figure 3:**
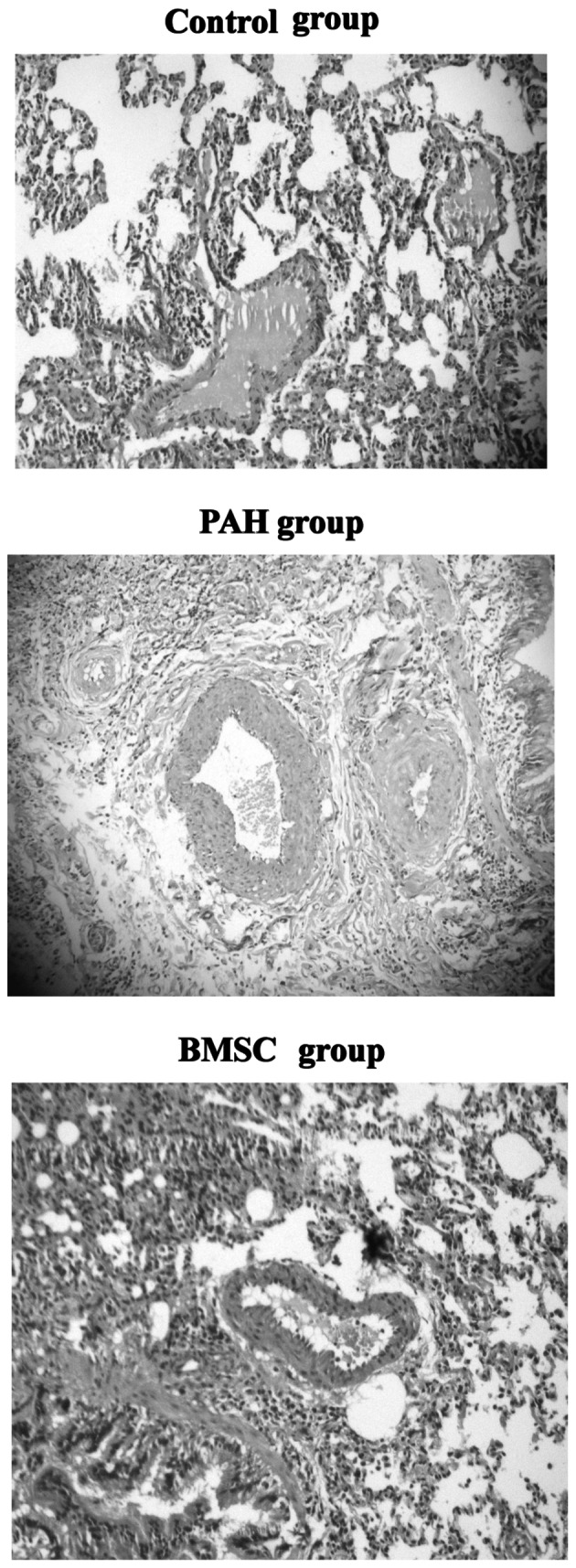
Effect of BMSCs on structural alterations in the pulmonary artery wall as determined by H&E staining 3 weeks after injection (x100 magnification). PAH, pulmonary arterial hypertension; BMSCs, bone marrow mesenchymal cells; H&E, hematoxylin and eosin.

H&E staining demonstrated that intima thickening and luminal stenosis were significantly decreased in the BMSC group compared with the PAH group. The MT% and VA% of muscular arteries was significantly improved in the BMSC group compared with the PAH group (P<0.05, Table IV, Fig. 3).

### Identification of the transplanted BMSCs

**Figure 4:**
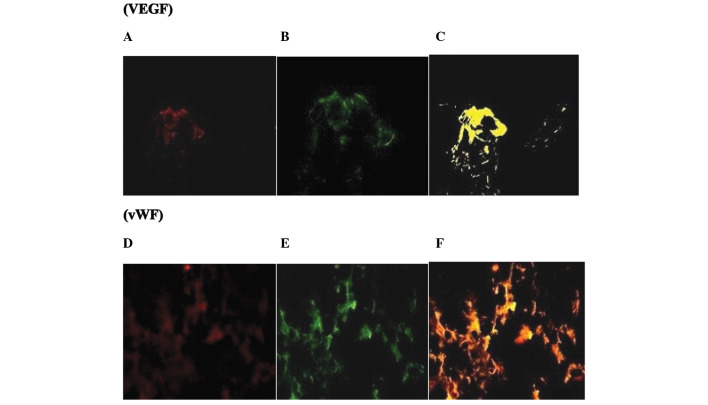
Identification of the transplanted BMSCs. (A and D) Transplanted BMSCs demonstrated red fluorescence, and (B amd E) sections stained with VEGF and vWF produced green fluorescence. (C and F) The merged images show yellow fluorescence (magnification x400). There was no evidence of red or green fluorescence in the control or PAH groups. VEGF, vascular endothelial growth factor; vWF, von Willebrand Factor; BMSCs, bone marrow mesenchymal cells.

In numerous regions, the red fluorescence-positive cells were observed coincident with the green fluorescence spots of VEGF and vWF antibody but not SP-C antibody, which suggested that the intravenous implantation of BMSCs resulted in their differentiation into vascular endothelial cells *in vivo* although they did not survive as lung cells. There was no evidence of red or green fluorescence in the control and PAH groups (Fig. 4).

## Discussion

PAH is a progressive disorder characterized by the progressive increase in pulmonary arterial pressure and resistance, eventually leading to right heart failure and mortality in patients with refractory disease. Although in the past ten years, the treatment of PAH has achieved apparent progress, the prognosis remains poor. Intravenous administration of drugs (e.g. prostacyclin, endothelin receptor antagonists) or nitric oxide inhalation may temporarily reduce PAH, but these effects are not lasting. In recent years, regeneration and gene therapy has become the research focus worldwide, however, stem cell research is still in its initial stages and so far there is no uniform method to treat PAH. Therefore, for further experimental studies, investigating reasonable and safe methods of treatment for PAH has become urgent.

BMSCs, multipotent progenitor cells derived from fetal bone marrow, could differentiate into distinctive end-stage cell types, including bone, cartilage, muscle, endothelial cells, vascular smooth muscle cells and other connective tissues. Studies have also demonstrated that BMSCs have the pluripotent ability to become endothelial progenitor cells and other cell lineages (15,16). BMSCs are able to secrete a variety of growth factors to promote angiogenesis, such as VEGF.

Transplantation of endothelial progenitor cells (EPCs) into the MCT-injured lung could repair the damage, but the treatment effect is not ideal. The studies on BMSC transplantation for pulmonary hypertension are limited. In our previous research (17), intravenous implantation of BMSCs improved the progression of RV impairment caused by MCT-induced PAH. The aim of this study was to further explore the effect of BMSCs on lung and heart impairment. First, we demonstrated that 2 weeks after sublingual vein administration of BMSCs to PAH rats, the pulmonary arterial pressure was significantly lower in the BMSC group compared with the nontreated PAH group. RVSP, MRVP and MPAP were significantly lower in the BMSC group compared with the PAH group, the ratio of RV/BW was significantly lower in the BMSC group compared with the PAH group. Notably, in the case of the present study, we found that the structural changes in the pulmonary vascular wall, such as VA and TAA, were significantly improved. Although the underlying mechanisms are complicated and not yet determined, several factors are expected to contribute, including the role of stem cell differentiation and para-secretion effects (3,18–23). Our experiments also demonstrated that the red fluorescence-positive cells were observed coincident with the green fluorescence spots of VEGF and vWF antibody but not SP-C antibody in numerous regions. These results suggest that the intravenous implantation of BMSCs results in the ability of these cells to differentiate into vascular endothelial cells *in vivo* but not lung cells. Therefore, transplantation of BMSCs by homing to the lung and transforming into vascular endothelial cells may create a wide range of collateral circulation, increase the total area of the pulmonary vascular bed, improve pulmonary blood supply and effectively reduce the pulmonary hypertension.

In conclusion, our results showed that intravenous implantation of BMSCs may improve not only the cardiac function and hemodynamics, but also the pulmonary vascular wall damage in PAH caused by MCT. Therefore, this study provides conslusive information for the use of this new method in the treatment of pulmonary arterial hypertension.

## Figures and Tables

**Table I:** Effect of MCT on hemodynamics and right ventricular weight one week after administration.

Variable	Control	PAH
RVSP (mmHg)	34.38±1.86	50.33±1.39^b^
MPAP (mmHg)	20.18±2.19	40.28±3.27^b^
MRVP (mmHg)	19.43±3.17	42.64±4.25^b^
RV/BW	0.49±0.03	0.57±0.06^b^

MCT, monocrotaline; PAH, pulmonary arterial hypertension; RVSP, right ventricular systolic pressure; MPAP, mean pulmonary arterial pressure; MRVP, mean right ventricular pressure; RV/BW, ratio of right ventricular to body weight.

a P<0.05,

b P<0.01,

c P<0.001 vs. control.

**Table II:** Effect of MCT on the pulmonary artery wall one week after administration.

Variable	Control	PAH
MT%	13.05±1.33	38.27±4.55^b^
VA%	44.09±4.17	33.39±6.63^b^

MCT, monocrotaline; PAH, pulmonary arterial hypertension. MT% = MT/ED and VA% = VA/TAA – MT, wall thickness; ED, blood vessel diameter; VA, pulmonary artery lumen area; TAA, vascular area.

a P<0.05,

b P<0.01,

c P<0.001 vs. control.

**Table III:** Effect of BMSCs on the hemodynamics and right ventricular weight 2 weeks after implantation.

Variable	Control	PAH	BMSCs
RVSP (mmHg)	35.17±1.86	56.84±1.54^b^	43.83±2.13^e^
MPAP (mmHg)	20.36±2.13	41.37±2.24^b^	26.82±3.42^e^
MRVP (mmHg)	19.74±5.23	41.68±6.17^b^	28.34±1.98^e^
RV/BW (%)	0.472±0.035	0.613±0.072^a^	0.547±0.041^d^

BMSCs, bone marrow mesenchymal cells; PAH, pulmonary arterial hypertension; RVSP, right ventricular systolic pressure; MPAP, mean pulmonary arterial pressure; MRVP, mean right ventricular pressure. RV/BW = ratio of right ventricular to body weight;

a P<0.05,

b P<0.01,

c P<0.001 vs. control;

d P<0.05,

e P<0.01 vs. PAH group.

**Table IV:** Effect of BMSCs on the pulmonary artery wall 2 weeks after implantation.

Variable	Control	PAH	BMSCs
MT%	12.08±1.30	45.21±4.37^b^	20.83±5.49^d^
VA%	42.31±4.39	20.36±6.81^b^	38.27±3.48^d^

BMSCs, bone marrow mesenchymal cells; PAH, pulmonary arterial hypertension. MT% = MT/ED and VA% = VA/TAA – MT, wall thickness; ED, blood vessel diameter; VA, pulmonary artery lumen area; TAA, vascular area.

a P<0.05,

b P<0.01,

c P<0.001 vs. control;

d P<0.05,

e P<0.01 vs. PAH group.
